# Clinical, Diagnostic, and Treatment Features of Body Packing in Brazil: Drugs, Cell Phones, and Beyond

**DOI:** 10.7759/cureus.27894

**Published:** 2022-08-11

**Authors:** Vinícius Henrique A Guimarães, Carolina Cassiano, Dylmadson Iago B Queiroz, Ricardo Pastore, Roberto Lenza, Carlo Jose Freire Oliveira

**Affiliations:** 1 Health Sciences, Federal University of Triangulo Mineiro, Uberaba, BRA; 2 Microbiology, Immunology, and Parasitology, Federal University of Triangulo Mineiro, Uberaba, BRA

**Keywords:** cellphone, prison health, drug concealment, drugs, body packing

## Abstract

Introduction: Body packing consists of the concealment of substances (drugs and non-narcotics) or products inside the human body with the purpose of smuggling and may represent an emergency due to the fatal risk of narcotic toxicity, intestinal obstruction, and visceral perforation. However, non-narcotic body packing, especially in developing countries, is under-evaluated. Thus, the objective of this study was to evaluate cases of body packers in Brazil as regards narcotic and non-narcotic contents.

Methods: This retrospective study analyzes the medical records of body packers admitted from January 2015 to December 2019 at one of the main tertiary hospitals in central Brazil.

Results: Ten cases of body packing were observed. We found that five patients carried drugs, while seven carried non-narcotic substances such as cell phones and accessories. All the patients were male, prisoners, and young adults. In six patients, there was gastrointestinal obstruction, and in three, there was acute narcotic intoxication. Abdominal radiography diagnosed eight of the cases. In nine of the cases, emergency laparotomy was required, but all patients successfully recovered.

Conclusion: There was a higher prevalence of body packing of non-narcotic content; however, diagnostic and surgical approaches were similar to those of narcotic content. Clinicians must be aware of both non-narcotic and narcotic body packing.

## Introduction

The strategy of illegally transporting drugs or other contents inside the human body is known as body packing. This practice tries to avoid law enforcement detection and is specially used while crossing borders between countries or entering prisons [1-3]. Individuals with contents hidden inside their bodies are known as body packers, and they usually conceal these items, wrapped in the form of capsules, by oral ingestion or rectal/vaginal insertion [4,5]. However, the packing of drugs inside the body poses a serious risk of acute narcotic toxicity because the packages can break and release the stored drug, which can be quickly absorbed by the digestive system [2,3].

Several studies have demonstrated the involvement of body packing in illegal drug trafficking, but body packing is more complex given the existence of its other face: the transport of non-narcotic substances. This non-narcotic content body packing side is less explored in the scientific literature [2,6]. Among these non-narcotic content types involved in body packing, cell phones and their accessories (i.e., chargers, chips, and batteries) should be highlighted [7,8]. Cell phones are a growing and serious problem for prison systems as they are used by prisoners to maintain contact with the outside world and execute criminal activities [9,10]. In Brazil, this scenario is also alarming, and the high prevalence of body packing by prisoners, visitors, and even corrupt jail employees to smuggle these cell phones and their accessories was demonstrated [11].

Despite the absence of the risk of acute narcotic toxicity in cases of body packing of non-narcotic objects, the risk of obstruction or perforation of the digestive system is also important to consider. Thus, in addition to being a serious public safety problem, non-narcotic content body packing can mean a serious emergency health condition [6-8]. Therefore, this study aimed to identify and describe cases of body packers treated in an emergency department in a tertiary hospital in the central region of Brazil. A previous preprint has been published [12].

## Materials and methods

This study was previously approved by the Research Ethics Committee (REC) of the Federal University of Triangulo Mineiro (UFTM), with project number Ethics Appreciation Presentation Certificate 32259220.7.0000.5154 and Authorization 4.225.774. A case series study was carried out among patients admitted between 2015 and 2019 in the Clinical Hospital of the Federal University of Triangulo Mineiro, which is one of the main tertiary hospitals in the state of Minas Gerais in the central region of Brazil. Patients were selected based on data from the hospital’s clinical management, as it is required by law for the hospital management to inform police authorities of all cases in which possible trafficked content is found during patient care. After selecting the patients, their medical files were reviewed to extract clinical, radiological, and surgical procedure data.

## Results

Ten cases of body packing were identified over the five-year study period in the hospital. The 10 patients were men (100%), and their average age was 29.9 years old (range: 19-46 years). All patients (100%) had already served some type of sentence in the prison system. Despite the similar profile of the patients, there were important variations in the contents ingested and the clinical, diagnostic, and treatment aspects of these body packers. Table 1 presents additional details about the cases.

**Table 1:** Clinical, diagnostic, and treatment aspects of the body packers. *It could not be determined whether the body packers did not specify the date they ingested the objects or whether there was an error in filling out the medical records. **UDE: upper digestive endoscopy

Case	Content	Time from ingestion to hospital	Signs and symptoms	Imaging examination	Content recovery	Treatment outcome
1	25 packs of marijuana	Unreported*	Emesis, chest pain, and chills	X-ray	UDE** and feces	Complete recovery
2	1 cell phone charger	Unreported*	Abdominal pain, hematemesis, and constipation	X-ray	Laparotomy and gastrostomy	Complete recovery
3	3 packs of cocaine	1 hour	Abdominal pain and paresthesia on the tongue	UDE**	Laparotomy and gastrostomy	Complete recovery
4	1 cell phone charger	5 months	Abdominal pain	X-ray	Laparotomy and gastrostomy	Complete recovery
5	1 cell phone	23 hours	Abdominal pain and emesis	X-ray	Laparotomy and gastrostomy	Complete recovery
6	1 Durepox, 1 pen-drive, 1 saw, 1 cell phone and its battery, and drug packs of cocaine and marijuana	Unreported*	Emesis	X-ray	Laparotomy and gastrostomy	Complete recovery
7	1 cell phone	37 days	None	X-ray	Laparotomy and gastrostomy	Complete recovery
8	1 cell phone and packs of crack	7 days	None	X-ray	Laparotomy, gastrostomy, and feces	Complete recovery
9	1 cell phone charger	8 days	Abdominal pain and emesis	Tomography	Laparotomy and gastrostomy	Complete recovery
10	31 packs with no identified drugs	Unreported*	Convulsion, decortication, and decreased levels of consciousness	X-ray	Laparotomy, gastrostomy, ileostomy, and colostomy	Complete recovery

In all the patients, body packing was carried out by ingestion. The types of content that were ingested varied; cell phones and accessories were present in 70% of the patients, drugs were found in 50% of the patients, and one patient (10%) ingested other objects. Two (20%) patients ingested both narcotic and non-narcotic objects. There were extreme variations in the time periods for which the substances were retained in the patients’ bodies before they were found, ranging from one hour to five months.

The signs and symptoms of the patients varied significantly. Of the patients, 60% had gastrointestinal symptoms such as emesis (40%), abdominal pain (50%), intestinal constipation (10%), hematemesis (10%), and chest pain (10%), which were ruled out as originating from other organs. In contrast, 20% of the patients reported no symptoms. Symptoms of acute narcotic intoxication were observed in 30% of the patients, which included chills (10%), paresthesia on the tongue (10%), convulsive crises (10%), decortication (10%), and decreased levels of consciousness (10%). In all these cases, rupture of the narcotic contents was subsequently confirmed.

**Figure 1:**
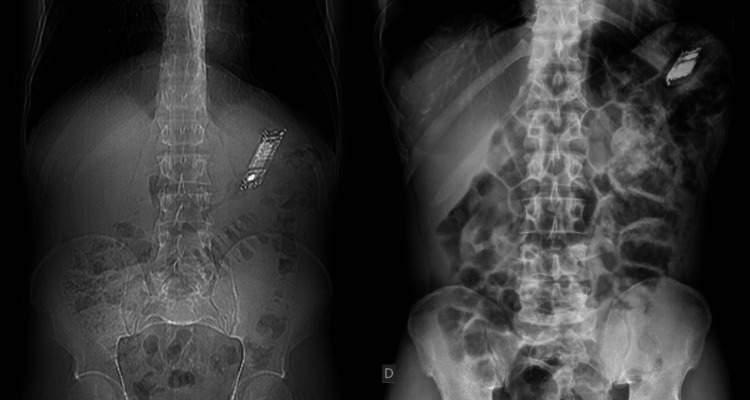
Radiographs showing the cell phones in the patients’ gastrointestinal tracts as obtained in the abdominal radiograph. In all these patients, the cell phones were clearly visible.

In 80% of the cases, the diagnosis was confirmed by imaging, more specifically abdominal radiography (Figure 1). Computed tomography (CT) was performed in 10% of the cases; however, the reason for this choice was not specified in the medical records. Upper digestive endoscopy (UDE) was performed in 10% of the cases.

**Figure 2:**
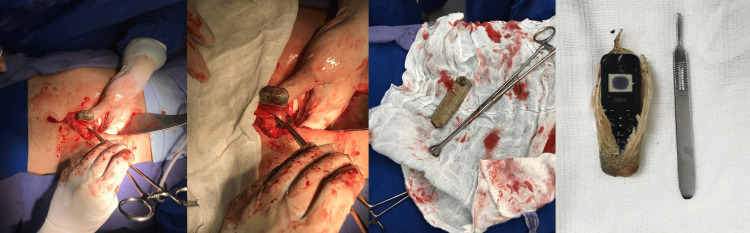
Intraoperative aspects of the cell phone extraction process from the gastrointestinal tract. A small cell phone was removed from the stomach by laparotomy access. The cell phone was also wrapped. The outer covering was removed, which exposed the small cell phone.

In 90% of the cases, the ingested contents, or parts of them, were retained in the stomach and failed to pass through the pyloric sphincter. In all these cases, an emergency surgical approach was necessary, and thus, exploratory laparotomy and gastrostomy were performed (Figure 2). In 10% of the cases, removal of the content occurred through UDE because part of the content was fixed in the lower esophageal sphincter. In 20% of the cases, the content had already passed the pylorus and was eliminated through the feces as these patients did not present with severe symptoms of acute narcotic intoxication.

**Figure 3:**
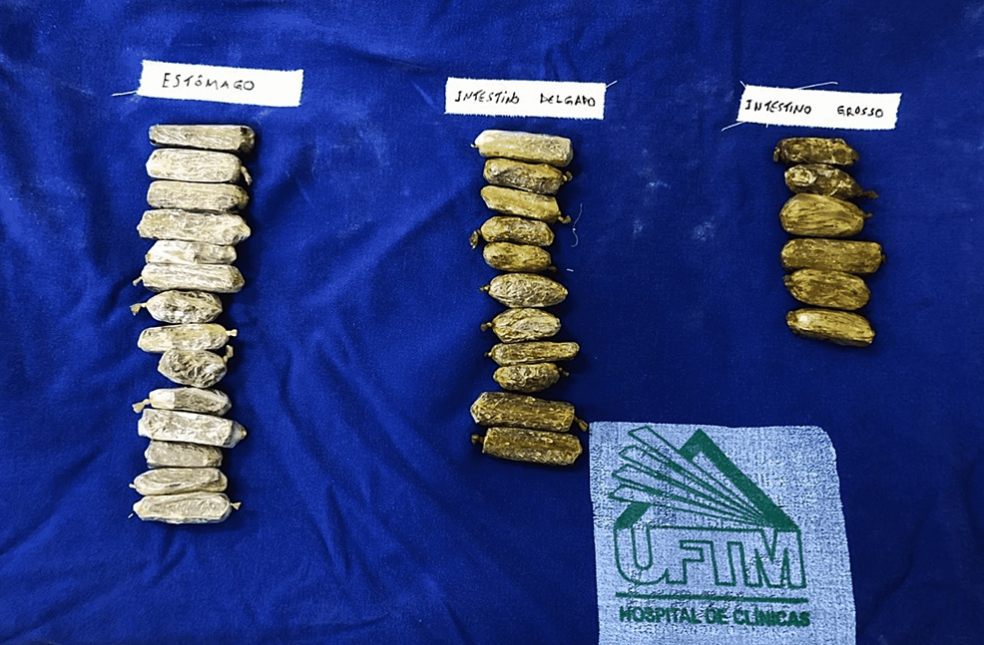
Characteristics of the drug packages after their removal from the patients involved in body packing. The different appearances of the packages indicate the different locations in which they were found within the gastrointestinal tract: on the left, packages from the stomach; in the middle, packages from the small intestine; and on the right, packages from the large intestine.

One (10%) patient presented with acute narcotic intoxication, and as the content already passed the pylorus, the patient was opted for ileotomy and colostomy to remove the content (Figure 3). In general, the treatment measures adopted were successful as there were no recorded deaths, with all the body packers recovered.

## Discussion

The body packers in our study were male young adults already in the prison system, who purposefully orally ingested foreign bodies. It is not known whether they were body packers or body stuffers. Our profile of young and male body packers matches that reported in other studies from several nations [13-17]. In addition, these patients ingested very different types of content. Non-narcotic content was predominant, which included cell phones and accessories (i.e., chargers and batteries), and accounted for 70% of the cases. Narcotic substances were ingested in 50% of the cases, which included marijuana, cocaine, and crack, which are compatible with the most found drugs in body packing cases in the literature [3].

Three of the five narcotic body packing cases experienced narcotic intoxication. Narcotic body packing can present asymptomatically until toxicity develops. Between these extreme situations, we could see patients with unspecific gastrointestinal or intoxication symptoms [1,3,18].

Numerous research on narcotic body packing has been published in the last decades, but non-narcotic body packing is less explored, especially in developing countries [2,6-8]. The findings of our study highlight a persistent problem that has recently emerged in Brazil and may partly help explain the origin of so many cell phones, accessories, and other non-narcotic contents in Brazilian prisons [11]. However, the problem of cell phones inside prisons is not only a challenge in Brazil but also an alarming global issue that has been reported in different countries such as the USA [9,10] and Kenya [10]. Thus, it is possible that body packing involving non-narcotic content can be a global problem that warrants more vigilant monitoring and investigation in other countries.

From a diagnostic point of view, the non-narcotic contents found in the patients in this study, especially cell phones and their accessories, were similar to the narcotic ones that were found. Prison authorities have already proven that these cell phones can be used by prisoners to plan escapes, threaten victims, extort, and maintain communication with criminal organizations. These communication tools are extremely desired by prisoners and are even used as currency within prisons [9-11].

Both the drug packs and non-narcotic objects that were ingested had similar clinical effects on the gastrointestinal tract, i.e., they caused similar symptoms such as constipation, abdominal pain, and emesis and even led to no presentation of symptoms in some cases [13]. However, only the narcotic substances showed acute narcotic intoxication. These symptoms can vary depending on the drug and the amount absorbed by the intestinal mucosa [13].

In the present study, we verified that reports of body packing involving non-narcotic and narcotic content can be easily visualized using simple abdominal radiographs. Cell phones, accessories, and drug packages are radiopaque; hence, radiographs and computed tomography (CT) scans are useful to confirm body packing and help determine the location of the content [1,8,13]. Due to the low radiation and cost but high availability, radiography has been reported to be the first examination required in suspected cases of body packing [8,13].

In this study, we observed a pattern in which the narcotic and non-narcotic contents affected the pyloric sphincter; therefore, in 90% of the cases, it was necessary to perform a laparotomy with gastrostomy to remove these contents from the gastrointestinal tract. It is also worth mentioning that laparotomy is an emergency surgical intervention in cases where the content is impacted and cannot be eliminated through the feces or there are clinical signs of acute intoxication, perforation, or obstruction. In the presence of symptoms, emergency surgery is a suitable option, while in their absence, elective surgery may be considered an option as some patients have gone on for several months without showing symptoms. Elimination through feces is a viable option if the patient remains monitored and radiological control is performed [3,13].

Finally, it is important to highlight that in Brazil, as in many countries, there are no national guidelines available; the body packing approach is based on widely agreed principles or centers guidelines where there are many cases [19]. Thus, research such as ours can share a perspective of how to deal with body packing cases, especially with non-narcotic content, which is under-evaluated in scientific literature.

## Conclusions

The narcotic body packing problem persists, but interestingly, more cases of body packing of non-narcotic contents were identified, especially cellphones. However, from a clinical, radiological, and therapeutic perspective, body packing of non-narcotic content approaches uses many similarities learned in the narcotic content studies. Both have intestinal symptoms, are easily diagnosed by X-rays, and can be resolved surgically. A high clinical suspicion is needed for potential non-narcotic and narcotic body packing mainly in patients with gastrointestinal symptoms coming from prisons.
